# Supplementary material for: Evaluation of the Diagnostic Accuracy of a New Biosensors-Based Rapid Diagnostic Test for the Point-Of-Care Diagnosis of Previous and Recent Dengue Infections in Malaysia
Source: Biosensors (Basel). 2021 Apr 22;11(5):129. doi: 10.3390/bios11050129 (PMC8143448; doi:10.3390/bios11050129)
Supplement: Supplementary file 1 [file biosensors-11-00129-s001.zip › biosensors-1137597-supplementary.pdf]

Supplement 1: Protocols of Reference Tests

S 1.1 Dengue IgG Indirect Enzyme-linked Immunosorbent Assay (ELISA)

The Panbio® Dengue IgG Indirect ELISA (Abbott, Illinois, USA) was used to detect IgG antibodies against dengue antigen serotypes 1, 2, 3 and 4 in serum for past exposure of dengue fever [1]. The test was performed following the manufacturer's instructions. Briefly, 100 µL of diluted serum sample was pipetted into the microwells, along with 1 well of Positive Control, 1 well of Negative Control and 3 wells of Calibrator. The plate was covered and incubated for 30 minutes at 37°C followed by a washing step of six times using diluted Wash Buffer. Then, 100 µL of horseradish peroxidase (HRP) Conjugated Anti-human IgG was added into each well and the plate was covered and incubated for 30 minutes at 37°C. The plate was then washed with diluted Wash Buffer for six times and 100 µL of TMB (3,3', 5,5;-tetramethylbenzidine) chromogen was added into each well. The plate was incubated at room temperature and 100 µL of Stop Solution was added after 10 minutes of incubation. The blue color developed from adding TMB chromogen was changed to yellow after the Stop Solution was added. The absorbance of each well was read within 30 minutes at a wavelength of 450 nm with a reference filter of 620 nm. For interpretation, the values for Positive Control, Negative Control and Calibrator was first validated based on the acceptable values provided in the kit specification sheet. A cut-off value was then determined by multiplying the average absorbance of the triplicates of the Calibrator and the calibrator factor provided in the kit specification sheet. An index value can be calculated by dividing the sample absorbance by the cut-off value and the Panbio Units can be calculated by multiplying the index value by 10. A sample with Panbio Unit of <9 was interpreted as IgG negative with no evidence of a past dengue infection. A sample with Panbio Unit of > 11 was interpreted as IgG positive with evidence of past or recent dengue infection. A sample with Panbio Unit of 9-11 was considered equivocal and to be retested where the second result was reported.

S 1.2 Dengue IgG Capture Enzyme-linked Immunosorbent Assay (ELISA)

The Panbio® Dengue IgG Capture ELISA (Abbott) was used to detect high-titer IgG antibodies against dengue antigen in patients with recent secondary dengue infection for serotypes 1, 2, 3 and 4 [2, 3]. The test was performed according to the manufacturer's instructions. In short, 100 µL of diluted serum was added into microwell coated with anti-human IgG antibodies, along with 1 well of Reactive control serum, 1 well of Negative control serum and 3 wells of Calibrator serum. The wells were covered and incubated for 1 hour at 37°C, followed by a washing step for six times with diluted wash buffer. Equal volume of horseradish peroxidase (HRP)-conjugated monoclonal antibody (Mab) is mixed with the reconstituted dengue virus antigens (serotypes 1-4), allowing the formation of antigen-MAb complexes. The antigen-MAb tracer solution (100 µL) was added into the wells and incubated for 1 hour at 37°C. The wells were then washed six times with diluted wash buffer before adding 100 µL of TMB solution and incubated at room temperature to allow color development. Stop solution was added after 10 minutes and the color changing of blue to yellow was observed. The absorbance of each well was read within 30 minutes at a wavelength of 450 nm, with a reference filter of 620 nm. The average absorbance of calibrator triplicates was calculated and multiply by the calibration factor to obtain the cut-off value. An index value was calculated by dividing the sample absorbance with the cut-off value, while the Panbio Units was then calculated by multiplying the index value by 10. A sample with Panbio Unit of <18 was interpreted as IgG negative with no detectable IgG antibody. A sample with Panbio Unit of >22 was interpreted as IgG positive with the presence of detectable IgG antibody that suggest active secondary dengue infection. A sample with Panbio Unit of 18-22 was considered equivocal and to be retested where the second result was reported.

S 1.3 Hemagglutination Inhibition (HI) Assay

The HI assay was performed according to the protocol described by Wang and Sekaran [4] based on the techniques recommended by Clarke and Casals [5]. Briefly, to prepare for the assay, serum sample was processed using acetone extraction method to remove any serum factors other than antibodies that may present to agglutinate the virus and goose red blood cell (GRBC). GRBC to be used in the assay was prepared in a range of concentrations by diluting with Dextrose Gelatin Veronal (DGV). Next, antigen hemagglutinating (HA) activity of a pool of antigens with seroptype 1, 2, 3 and 4 was determined to calculate the dilution needed to obtain an antigen concentration of 8 HA units. The diluted antigen was back titrated to ensure consistency and the confirmed dilution was used throughout the assay. For the HI assay, 25 μL of 0.4% bovine albumin borate saline (BABS) was added from Well 2 to Well 12 in a 96-well V-bottom microtiter plate followed by 50 μL and 25 μL of the extracted serum sample to Well 1 and Well 12, respectively. The serum was then titrated from Well 1 to Well 11 by pipetting 25 μL of the solution from one well to the next. Next, 25 μL of the diluted antigen was added from Well 1 to Well 11, followed by 25 μL 0.4% BABS to Well 12 as the serum control. The plate was covered and incubated for 24 hours at 4°C. Freshly prepared GRBC in a volume of 50 μL was added into all wells and the plate was read after incubation of 45 mins at 37°C. The results are reported as the reciprocal of the antibody titer based on the highest serum dilution that prevent the antigen from agglutinating the GRBC. Antibody titer of <1:10 indicated no detectable antibody and was interpreted as negative result, while antibody titer of ≥1:10 indicated the presence of neutralizing antibodies against dengue antigen and was considered as a positive result [6].

S 1.4 Focus Reduction Neutralization Test (FRNT)

FRNT was performed by adapting the protocol of plaque reduction neutralization test (PRNT) recommended by WHO [7] using modified immunostaining method described by Ngwe Tun, Muta [8]. FRNT of each serum sample was performed against 4 dengue serotypes, DENV-1 (WP), DENV-2 (NGC), DENV-3 (S78) and DENV-4 (VP), and was reported individually. First, serum sample was heat inactivated for 30 minutes at 56°C and was serially diluted fourfold with 2% fetal bovine serum (FBS) in Dulbecco's Modified Eagle Media (DMEM) () up to the dilution of 1:10240. Next, 150 μL of each diluted serum sample was mixed with an equal volume of each serotype of DENV which contained 60 focus-forming units. The mixture was incubated for 1 hour at 37°C and was inoculated onto a monolayer of Vero CCL-81 () cell in a 96-well cell culture plate for 1 hour at 37°C. The infected cells were then overlaid with 1.25% methylcellulose in 2% FBS in DMEM. The plate was incubated for 3 days at 37°C before immunostaining. The immunostaining procedure started with a washing step of 3 times with phosphate-buffered saline (PBS), followed by fixing the plate with 300 μL 4% formaldehyde in PBS for 30 minutes at room temperature. The plate was then washed with PBS and the cells were fixed by 1% NP-40 in PBS for 15 minutes at room temperature. The wells were washed and were blocked with 3% skim milk in PBS for 2 hours at room temperature. After washing, the cells were treated with a primary anti-flavivirus group antigen mouse monoclonal IgG2a IgG1 (Merck Millipore, Darmstadt, Germany) at a dilution of 1:500 in 1% skim milk for 1 hour at 37°C. Next, the cells were washed and treated with secondary goat anti-mouse IgG peroxidase conjugate (Merck Millipore) at a dilution of 1:500 in 1% skim milk for 1 hour at 37°C. Lastly, the wells were washed and stained with 0.5mg/mL of 3,3'-Diaminobenzidine (DAB) for 20 minutes in dark. After washing with purified water, the number of foci per well was counted under a microscope. FRNT95 titer was reported as the reciprocal of the highest serum dilution that presented a 95% or greater reduction in the mean number of foci from the triplicates in relative to the control wells that contained no serum. A monotypic antibody response towards one serotype was reported when the neutralization titer of a sample was ≥1:10 to only one serotype. For a sample with neutralization titer of ≥1:10 for more than one serotype, it was reported as a multitypic antibody response, where the serotype with the highest FRNT95 titer is

assumed to be the current infecting serotype. A negative result was reported when the neutralization titers of a sample were <1:10 towards all serotypes [9, 10].

References

1. Schüttoff, T., et al., *Enhancing the concordance of two commercial dengue IgG ELISAs by exchange of the calibrator sample.* J Clin Virol, 2019. **118**: p. 1-5.
2. Blacksell, S.D., et al., *Comparison of seven commercial antigen and antibody enzyme-linked immunosorbent assays for detection of acute dengue infection.* Clin Vaccine Immunol, 2012. **19**(5): p. 804-10.
3. Vazquez, S., et al., *Evaluation of immunoglobulin M and G capture enzyme-linked immunosorbent assay Panbio kits for diagnostic dengue infections.* J Clin Virol, 2007. **39**(3): p. 194-8.
4. Wang, S.M. and S.D. Sekaran, *Early diagnosis of Dengue infection using a commercial Dengue Duo rapid test kit for the detection of NS1, IGM, and IGG.* The American journal of tropical medicine and hygiene, 2010. **83**(3): p. 690-695.
5. Clarke, D.H. and J. Casals, *Techniques for hemagglutination and hemagglutination-inhibition with arthropod-borne viruses.* Am J Trop Med Hyg, 1958. **7**(5): p. 561-73.
6. Lukman, N., et al., *Comparison of the Hemagglutination Inhibition Test and IgG ELISA in Categorizing Primary and Secondary Dengue Infections Based on the Plaque Reduction Neutralization Test.* BioMed research international, 2016. **2016**: p. 5253842-5253842.
7. WHO, *Guidelines for plaque reduction neutralization testing of human antibodies to dengue viruses*. 2007, World Health Organization: Geneva.
8. Ngwe Tun, M.M., et al., *Persistence of Neutralizing Antibody Against Dengue Virus 2 After 70 Years from Infection in Nagasaki.* BioResearch open access, 2016. **5**(1): p. 188-191.
9. Endy, T.P., et al., *Relationship of preexisting dengue virus (DV) neutralizing antibody levels to viremia and severity of disease in a prospective cohort study of DV infection in Thailand.* J Infect Dis, 2004. **189**(6): p. 990-1000.
10. Ngwe Tun, M.M., et al., *Serological characterization of dengue virus infections observed among dengue hemorrhagic fever/dengue shock syndrome cases in upper Myanmar.* J Med Virol, 2013. **85**(7): p. 1258-66.
